# Generation of attosecond electron packets via conical surface plasmon electron acceleration

**DOI:** 10.1038/srep19056

**Published:** 2016-01-14

**Authors:** S. R. Greig, A. Y. Elezzabi

**Affiliations:** 1 Ultrafast Optics and Nanophotonics Laboratory Department of Electrical and Computer Engineering University of Alberta Edmonton, AB T6G 1H9 Canada

## Abstract

We present a method for the generation of high kinetic energy attosecond electron packets via magnetostatic and aperture filtering of conical surface plasmon (SP) accelerated electrons. The conical SP waves are excited by coupling an ultrafast radially polarized laser beam to a conical silica lens coated with an Ag film. Electromagnetic and particle tracking models are employed to characterize the ultrafast electron packets.

Ultrafast electron sources open the way for real-time imaging of fundamental physical, chemical, and biological processes with sub-nanometer and sub-picosecond resolution. Typical ultrafast electron based techniques include ultrafast electron diffraction (UED)^1^,^2^, and ultrafast transmission electron microscopy (UTEM)^2^,^3^. There exists a variety of methods for generating the necessary ultrafast electron packets, including photoemission from a photocathode illuminated by an ultrafast laser pulse^1^,^2^,^3^, photoemission from nanotips^4^,^5^,^6^, and photoemission followed by subsequent ponderomotive acceleration in intense surface plasmon (SP) fields^7^,^8^,^9^,^10^. Recently, there has been increased interest in SP based platforms as they have enabled the generation of high energy femtosecond electron packets^8^, and can employ a multitude of geometries including prisms^7^,^8^,^9^,^10^,^11^, nanotips^12^, nanoantennas^13^,^14^, and gratings^15^. In order to reach keV kinetic energies, these platforms typically require the use of complex laser amplifier systems. While the SP fields are excited with ultrafast laser pulses (<100 fs), the electron packets generated possess a broad range of kinetic energies^7^, which undermines the ultrafast packet duration due to the electrons dispersing in time as they propagate through free space. With the increased push towards generation of attosecond electron packets, this free space time dispersion becomes a dominant factor in determining the final duration of the electron packet^16^.

A few techniques have been proposed to overcome this dispersion and allow generation of ultrashort electron packets^17^,^18^,^19^. Baum *et al.* suggested a method to chirp the electron packet such that the packet will compress itself in time as it propagates through free space^20^. Ultimately, the greatest challenge in generating a short electron packet that does not disperse in time is achieving a low spread in the kinetic energy spectrum. Thus, all of the electrons will travel at the same velocity. This task has proven to be quite difficult to achieve through photogeneration and ponderomotive acceleration of the electron packets. This is due to the fact that such electrons experience spatially and temporally nonuniform accelerating electric fields. Overcoming this limitation requires either filtering or control of the kinetic energy of the electrons within the generated packet. One proposed technique for controlling and tuning the kinetic energy of SP generated electrons is through the introduction of single cycle terahertz (THz) pulses to influence the electrons’ interaction with the SP field^21^,^22^. Additionally, gating of the electron packet can also be achieved through implementation of electro-static filtering^23^. The spread of electron kinetic energies can also be controlled by limiting the electron emission region with a dielectric aperture deposited directly on the metallic film^24^.

In this work, we present a design of an ultrafast, high energy, conical lens based nanoplasmonic electron source. By harnessing the unique focusing property of radially polarized light, and magnetostatic spatial filtering, extremely short electron packets of attosecond durations and keV kinetic energies are produced. We show that by varying the magnetic field strength and the parameters of a physical aperture, electron packets with various kinetic energies and temporal packet lengths can be tailored to a specific need. Furthermore, the keV kinetic energies are achieved with laser pulse energies achievable from a simple laser oscillator. This electron source demonstrates a new paradigm in ultracompact, high energy, attosecond electron source development for time-resolved UED and UTEM.

## Electron acceleration via conical SP waves

**Figure 1:**
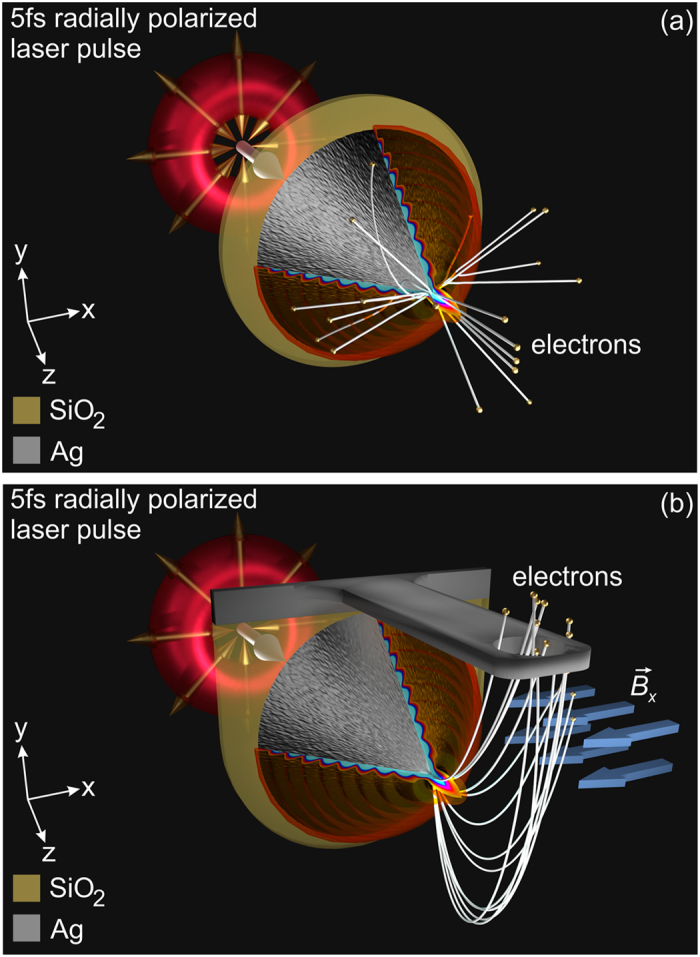
Schematic depiction of a nanoplasmonic attosecond electron source consisting of a 50 nm Ag film deposited on a conical silica lens. The electrons are generated by a 5 fs radially polarized laser pulse with a central wavelength of 800 nm and are accelerated by the nanoplasmonic field. (**a**) Configuration in the absence of a static magnetic field, where the electron packet generated has no defined direction, as shown by an ensemble of electron trajectories. (**b**) Configuration in the presence of a static magnetic field at 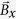 = −1 T. Here, the electron trajectories show that the generated electron packet is now directed towards the detector placed 50 μm away and pass through the aperture for energy filtering.

**Figure 2:**
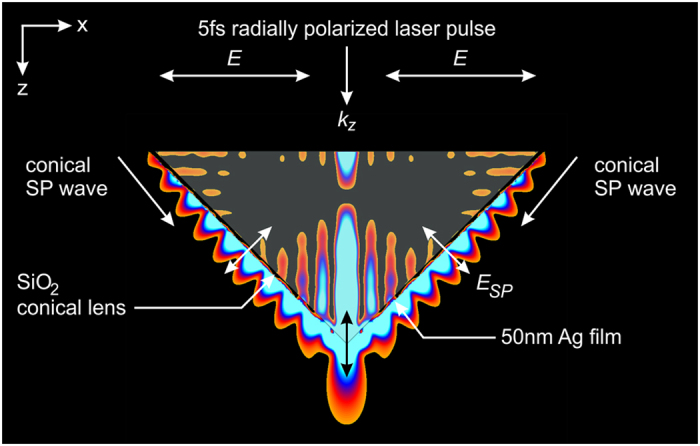
Two dimensional slice of the electric field intensity profile of the conical SP wave on the Ag coated conical lens when excited by a 5 fs radially polarized laser pulse with electric field strength *E*. The two-headed arrows represent the electric field oscillation direction of the laser field, *E*, and the SP field, *E*_*SP*_.

The geometry used for generating the keV attosecond electron packets is depicted in Fig. 1. The electron source consists of a 50 nm Ag film deposited on a conical silica lens with a diameter of 10 μm, an apex angle of 91°, and a refractive index *n = *1.45. To ensure that the findings are generalizable to a realistic situation, the Ag film is modelled to have a surface roughness of 2.8 nm rms (Ref. 25) such that the influence of enhanced SP fields localized at random spots is captured. A traveling SP wave is excited by a λ = 800 nm radially polarized laser pulse with pulse duration of τ = 5 fs and an electric field strength, *E*. It should be noted that all values of *E* refer to the peak value of the incident field. The use of a radially polarized beam affords the benefit of having the electric field polarization oriented such that it is p-polarized with respect to the Ag film along the entire azimuthal angle of the conical lens. This leads to the excitation of traveling SP waves encompassing the entire conical surface of the Ag film, a so-called conical SP wave. Consequently, this results in a greater peak enhancement of the electric field (63 times) at the apex than for the case of linear polarization (11 times). Figure 2 depicts the electric field intensity profile when the tip is excited by a radially polarized 5 fs laser pulse. The high intensity electric fields that are present within the SiO_2_ lens itself are due to the interference of out-of-plane reflections at the glass-metal interface along the entire azimuthal angle of the lens. As the SP wave propagates along the Ag film, free electrons are ejected through a three-photon absorption process^11^ from the film and are able to interact with the SP electric field, *E*_*SP*_. This interaction results in two sources of kinetic energy gain, ponderomotive and non-ponderomotive. In the ponderomotive interaction, electrons experience a significant difference in electric field strength on the positive and negative half-cycles. Thus, over multiple cycles, the electron is accelerated or decelerated, dependent on the polarity of the *E*_*SP*_ field. However, for short pulse durations, there is additional energy gained by non-ponderomotive acceleration due to the SP field^15^. This energy gain occurs over less than an oscillation cycle and depends on the phase of the SP field that the electron experiences when it is generated.

The employed model consists of two stages: first, the electric and magnetic fields are computed through numerical calculation of Maxwell’s equations utilizing the finite-difference-time-domain (FDTD) method; second, electrons are generated by placing them randomly (in space and time) along the vacuum side of the Ag film. Once generated, these electrons are subsequently tracked in time and space as they interact with the *E*_*SP*_ field through the nonrelativistic equation of motion. As the electrons are generated via a three-photon process, each electron is statistically weighted to be proportional to the cube of the electric field intensity at its spatial and temporal generation location. To verify that no tunneling emission will occur at the electric field strengths used, the Keldysh parameter, 
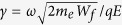
, is calculated, where *ω* is the laser frequency, *m*_*e*_ is the mass of the electron, *W*_*f*_ is the work function of Ag, and *q* is the electron charge. For 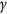 > 1, multi-photon ionization will dominate, while for 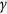 < 1, electron tunneling emission will dominate. For our operating parameters, and with the average surface enhancement of 3, a value of 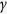 = 1.83 is calculated, indicating that electron emission will occur through a multi-photon process. Throughout the calculations, the peak intensity of the optical pulse is kept below 1.2 × 10^12^ W/cm^2^ which is below the experimentally determined damage threshold of a thin Ag film on a silica prism^25^. At this intensity, and with a spot size diameter of ~6 μm for a radially polarized beam, the corresponding energy per laser pulse is calculated to be ~1 nJ. Such a low laser pulse energy can be achieved with a typical ultrafast laser oscillator. It is estimated that at the highest intensities considered (1.2 × 10^12^ W/cm^2^), there will be ~10^4^ electrons/packet^25^. However, after passing through the aperture, the number is significantly reduced to 4% of its initial value. As this value is well below 10^7^ electrons/packet, space charge effects will not be encountered^25^.

## Results & Discussion

**Figure 3:**
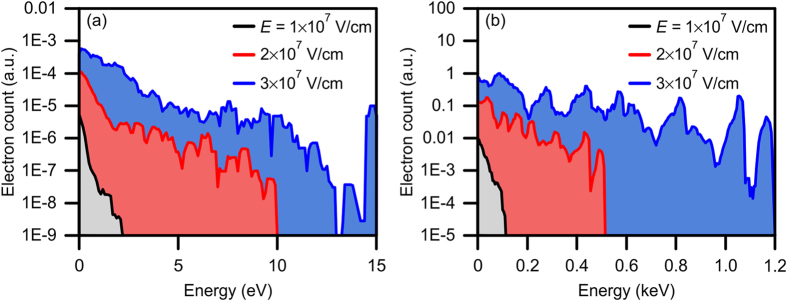
Electron kinetic energy spectra for an excitation with (**a**) linear polarization and (**b**) radial polarization. The electron count scale is normalized to the radial polarization configuration at *E* = 3 × 10^7^ V/cm. Note the difference in the electron count and kinetic energy scales between (**a**,**b**).

The kinetic energy spectra for a linearly polarized excitation laser pulse at various values of *E* are depicted in Fig. 3(a). At the maximum *E* = 3 × 10^7^ V/cm a maximum electron kinetic energy of 15 eV is achieved, with the majority of electrons (85%) being accelerated to energies below 2 eV. Lowering *E* shifts the electron kinetic energy spectrum towards lower energies with a maximum at 2.2 eV, achieved at *E* = 1 × 10^7^ V/cm. Clearly, utilizing linearly polarized laser pulses at these field strengths limits the electron’s energy to only a few eVs. The poor performance of the linearly polarized configuration can be directly attributed to the laser pulse only coupling to an SP wave in one plane (i.e. the *xz* plane at the center of the conical lens in Fig. 1(a)), leading to a reduction in the electric field enhancement occurring at the apex of the conical lens.

In order to increase the maximum attainable kinetic energy of the generated electrons, the incident laser pulse can be made to be radially polarized. As discussed earlier, this leads to conical SP waves that are directed towards and focused at the tip where they constructively interfere. Therefore, a peak electric field enhancement of 63 × is achieved at the tip, compared to 11 × for the case of linear polarization.

The electron kinetic energy spectra for excitation with a radially polarized laser pulse at various *E* are depicted in Fig. 3(b). Owing to the increased electric field enhancement from the radially polarized configuration, a maximum kinetic energy of 1.2 keV can be achieved at *E* = 3 × 10^7^ V/cm, 80 times higher than for excitation with linear polarization at the same electric field strength. Remarkably, at this excitation field strength, a large number of electrons (45% of the total electrons) have kinetic energies above 0.25 keV. The maximum kinetic energy drops to 0.11 keV at *E* = 1 × 10^7^ V/cm. In addition to the increased kinetic energy of the generated electrons, employing a radially polarized configuration greatly increases the total number of photogenerated electrons. This is evidenced by comparing the electron counts between Fig. 3(a,b), where the maximum total number of electrons for the radial polarization is ~4,300 times that of the linear case at *E* = 3 × 10^7^ V/cm. This can be attributed to excitation of the conical SP waves along the entire conical surface of the Ag film.

**Figure 4:**
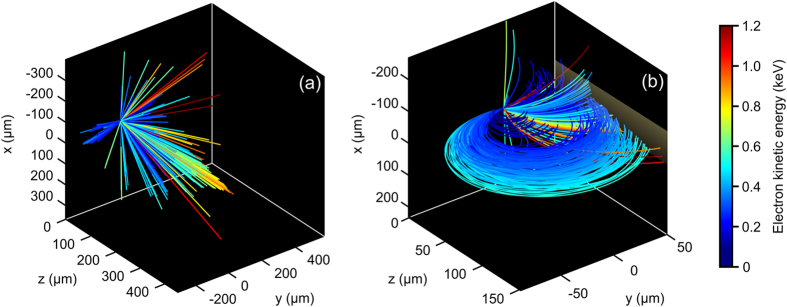
Representative electron trajectories (**a**) in the absence of the magnetostatic field, and (**b**) with an applied 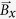 = −1 T. The color scale represents the electron kinetic energy.

From the broad kinetic energy spectra present in Fig. 3, it can be ascertained that any electron packet generated from either configuration (linear or radial polarization) would result in an extremely long packet duration due to the inherent time dispersion. Therefore, it is of paramount importance to implement a mechanism that can either tune or filter the kinetic energies of the electrons in order to produce electron packets with a minimal spread in kinetic energy. One such configuration that is investigated here is kinetic energy filtering of the electrons through the introduction of a static magnetic field. In this manner, the accelerated electrons will be directed away from the tip towards a detector placed in the +*y* direction (Fig. 1(b)) and spatially dispersed in accordance to their kinetic energy. The introduction of the magnetic field causes the electrons to travel in a circular trajectory with a radius of 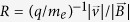, where 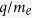 is the charge-to-mass ratio of the electron, 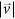 is the electron velocity, and 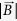 is the magnetic field strength. Since the radius is proportional to the velocity, and hence the kinetic energy of the electron, the electrons will spread out along the *z* axis of the detector. A sample of 500 electron trajectories in the absence of any external magnetic field is shown in Fig. 4(a). Since the accelerating force acts in both the outward radial and the longitudinal directions, the accelerated electrons are spread accordingly. While the generated electrons have keVs of kinetic energy, the lack of directionality in this arrangement makes it inadequate for UED and UTEM applications. Conversely, applying a magnetic field breaks the symmetry of the trajectory of the electrons being accelerated via the conical SP wave. Figure 4(b) illustrates such action, where the electrons are now directed in one direction (towards the detector) along a circular, velocity-dependent path.

**Figure 5:**
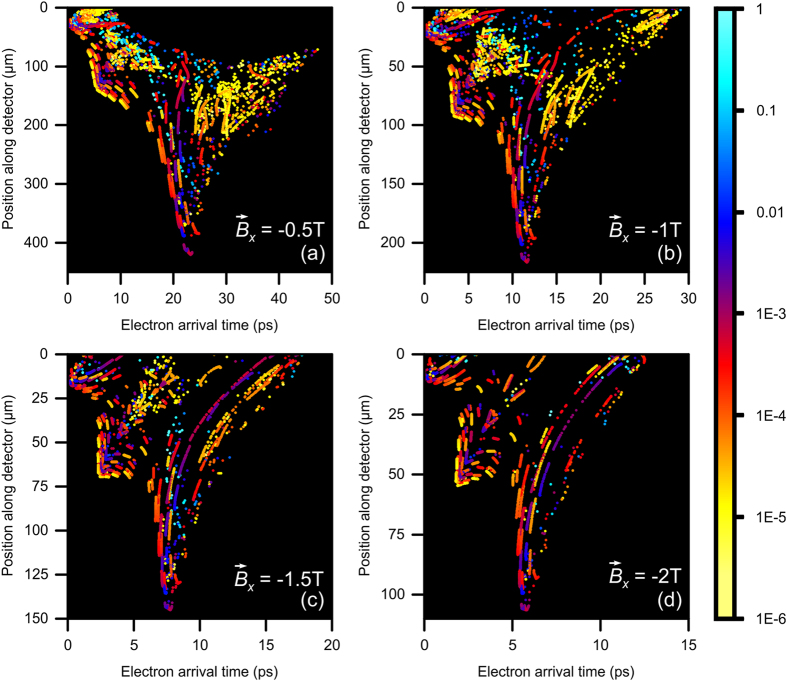
Electron arrival time as a function of position along a detector placed 50 μm away from the tip, for *E* = 3 × 10^7^ V/cm and with an applied static magnetic field 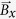
* = *(**a**) −0.5 T, (**b**) −1 T, (**c**) −1.5 T, and (**d**) −2 T. The color scale represents the normalized logarithmic electron count. Note the difference in axis scales.

Varying the strength of the applied magnetic field results in the tuning of the packet length of the generated electron packet and offers a scheme for accessing the attosecond regime. This can be illustrated with the following representative arrangement, where the detector is placed 50μm away from the tip in the +*y* direction, *E* is fixed at 3 × 10^7^ V/cm, and 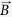 is directed in the –*x* direction. Figure 5 depicts a 2-dimensional map of the arrival time of the electrons as a function of their final position along the *z*-axis of the detector for 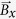 = −0.5 T, −1 T, −1.5 T, and −2 T, with the color representing the normalized electron count on a logarithmic scale. The spatial spreading afforded by the magnetic field can distinctly be seen in Fig. 5 where the electrons spread out in space as they reach the detector. Here, electrons arrive up to 420 μm away from the tip for 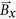 = −0.5 T. For increasing 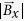, electrons will arrive at the detector closer to *z* = 0, thus reducing the spatial spreading of the packet. Additionally, the inherent time dispersion of the electron packet is evident in Fig. 5. In the case of 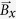 = −0.5 T, electrons can take 47 ps to arrive at the detector, but only 12 ps for 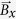 = −2 T. Notably, at a given 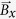, there are specific locations along the detector’s surface where the duration of the electron packets is <1 ps, and others where the duration is extremely long (>10 ps). The reason for the wide spread in the electron packet durations is due to the fact that each electron experiences a different spatial and temporal electric field strength as it gets accelerated within the SP field. That is, the SP acceleration process is sensitive to the local magnitude of the SP field and the phase of the generation of the electron with respect to the phase of the conical SP wave. When the magnetic field is introduced, it acts to curve the trajectory of the electrons in a circular fashion where their initial trajectory ultimately dictates where and when they will arrive at the detector. Each electron will have its velocity oriented in a different direction based on its specific interaction with the SP field. As such, electrons will arrive at the detector at different locations, resulting in the spread of both kinetic energy and arrival time at a distinct position along the detector. Clearly, the introduction of a static magnetic field helps in filtering the electrons to achieve ultrashort electron packets.

To spatially gate the electrons at the detector, an aperture is placed in front of the detector (depicted in Fig. 1(b)). This opening acts to define the position along the detector that is exposed to electrons, as well as, the arrival angles of the electrons. By varying the diameter (*d*), thickness (*t*), and location (*l*) of the aperture, the kinetic energy and the packet length of the electron packet can be controlled. Through predetermined selection of these parameters, the electron packet can be tailored to the specific needs for a given application.

**Figure 6:**
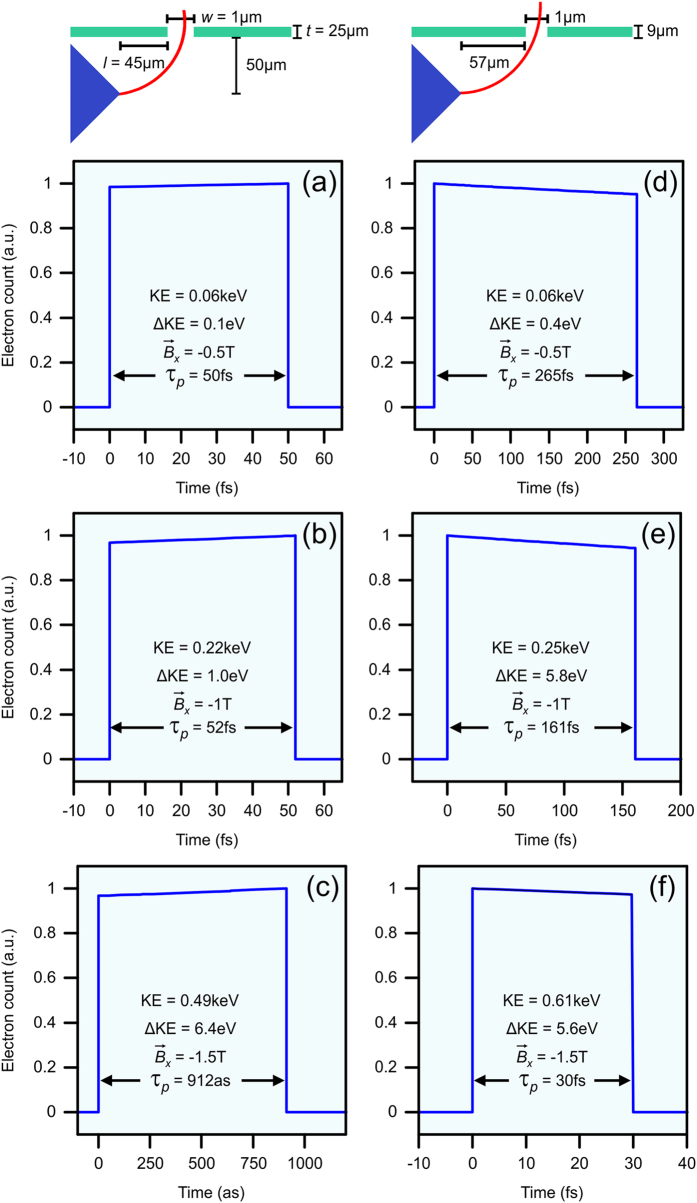
Electron packet duration at a detector placed 50 μm away from the tip, with schematic depictions of aperture parameters used at: 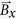 = (**a**) −0.5 T, (**b**) −1 T, and (**c**) −1.5 T for aperture parameters *d* = 1 μm, *t* = 25 μm, and *l* = 45 μm; 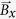 = (**d**) −0.5 T, (**e**) −1 T, and (**f**) −1.5 T for aperture parameters *d* = 1 μm, *t* = 9 μm, and *l* = 57 μm. KE is the weighted central kinetic energy, ΔKE is the kinetic energy spread, and 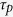 is the temporal duration of the electron packet. Note that the schematic depictions of the aperture parameters are not to scale.

The key characteristics defining an ultrafast electron packet are the temporal length (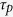), and the weighted central kinetic energy (KE). Figure 6 depicts electron packet arrival times chosen for two illustrative sets of aperture parameters and for 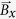 = −0.5 T, −1 T, and −1.5 T with the corresponding 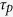 and KE, labelled on the respective electron packets. The kinetic energy spread (ΔKE) has also been included for completeness. Choosing the aperture thickness to be 25 μm, the diameter to be 1 μm, and locating it 45 μm from the tip, we obtain electron packets with kinetic energies varying from 0.06 keV at 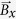 = −0.5 T (Fig. 6(a)) to 0.49 keV at 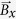 = −1.5 T (Fig. 6(c)). For 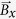 = −0.5 T, the packet length is 50 fs, while a packet length of 52 fs is achieved for 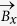 = −1 T. Notably, an ultrashort packet length of 912 as is observed at 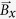 = −1.5 T. The characteristic square shape of the generated electron packets depicted in Fig. 6 arises due to the magnetic field acting to effectively map electrons from a location on the tip to a position along the detector based on their interaction with the SP field. As such, the electrons within the detected packet will have experienced similar acceleration due to the localized SP field (i.e. resulting in similar kinetic energies and electron counts). Selecting a different set of aperture parameters (*d* = 1 μm, *t* = 9 μm, *l* = 57 μm) results in increased kinetic energies, albeit with longer duration packets, as shown in Fig. 6(d–f). Figure 6(d) depicts the 0.06 keV, 265 fs electron packet which is observed for 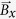 = −0.5 T. Varying 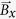
to −1 T or −1.5 T results in 0.25 keV, 161 fs and 0.61 keV, 30 fs electron packets, respectively. The generated electron packets exit the aperture with central angles, *θ*, ranging from −8.19° to 5.69° with angular spreads, *Δθ*, from 0.01° to 0.93° (Table 1). It should be noted that the two sets of aperture parameters discussed above are chosen as illustrative examples and that electron packets with various desired characteristics can be chosen by modifying the parameters of the aperture.

**Figure 7:**
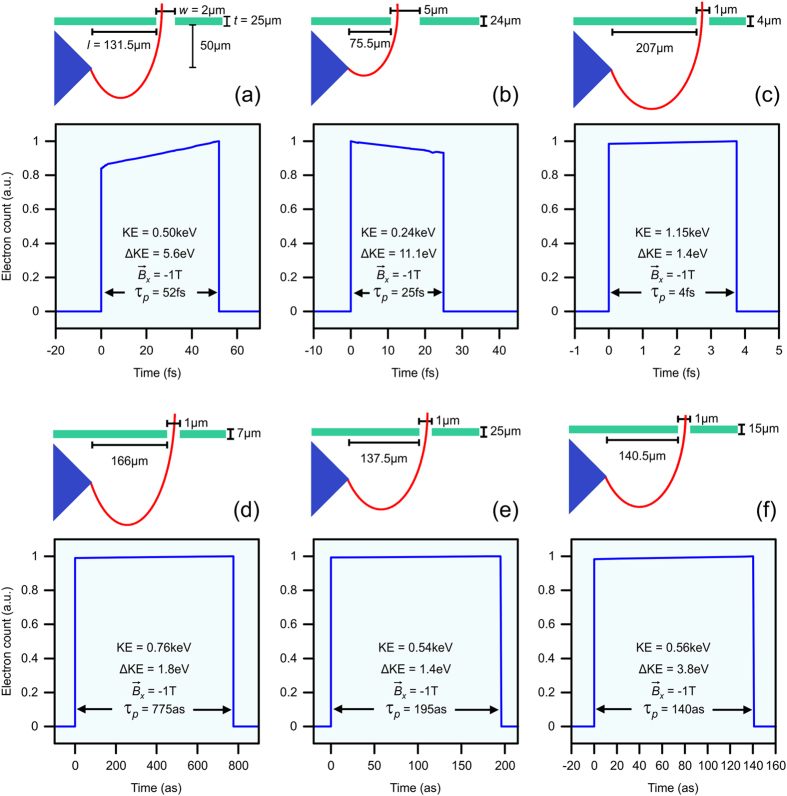
Electron packet duration at a detector placed 50 μm away from the tip, at a fixed *E* = 3 × 10^7^ V/cm and 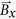 = −1 T with aperture parameters (**a**) *d* = 2 μm, *t* = 25 μm, *l* = 131.5 μm, (**b**) *d* = 5 μm, *t* = 24 μm, *l* = 75.5 μm, (**c**) *d* = 1 μm, *t* = 4 μm, *l* = 207 μm, (**d**) *d* = 1 μm, *t* = 7 μm, *l* = 166 μm, (**e**) *d* = 1 μm, *t* = 25 μm, *l* = 137.5 μm, and (**f**) *d* = 1 μm, *t* = 15 μm, *l* = 140.5 μm. KE is the weighted central kinetic energy, ΔKE is the kinetic energy spread, and 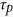 is the temporal duration of the electron packet. Note that the schematic depictions of the aperture parameters above each electron packet are not to scale.

As increasing 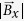 leads to a reduction in the spatial spreading of the electrons along the length of the detector, it becomes more challenging to select aperture parameters that result in desirable electron packets at various magnetic field strengths. Therefore, it is also possible to fix 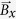 and examine different aperture parameters to achieve various electron packets. Figure 7(a–c) depict three illustrative femtosecond electron packets arriving at the detector for different aperture parameters at a fixed 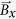 = −1 T. For *d* = 2 μm, *t* = 25 μm, and *l* = 131.5 μm, an electron packet with KE = 0.50 keV, and 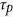 = 52 fs is observed (Fig. 7(a)). With *d* = 5 μm, *t* = 24 μm, and *l* = 75.5 μm, an electron packet with KE = 0.24 keV and 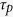 = 25 fs is achieved (Fig. 7(b)). An ultrashort, high energy electron packet with KE = 1.15 keV and 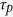 = 4 fs is achieved for *d* = 1 μm, *t* = 4 μm, and *l* = 207 μm (Fig. 7(c)). Remarkably, attosecond electron packets with kinetic energies >0.5 keV (Fig. 7(d–f)) can be achieved through proper choice of the aperture parameters. With *d* = 1 μm, *t* = 7 μm and *l* = 166 μm a high energy (KE = 0.76 keV) attosecond (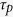 = 775 as) packet can be achieved. An electron packet with 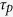 = 195 as and KE = 0.54 keV can be achieved with *d* = 1 μm, *t* = 25 μm and *l* = 137.5 μm. Notably, an ultrashort electron packet with 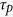 = 140 as packet and KE = 0.56 keV is observed for the aperture parameters *d* = 1 μm, *t* = 15 μm and *l* = 140.5 μm. Again, the electron packet characteristics can be tuned for a specific need by tailoring the aperture parameters.

## Conclusion

In conclusion, we have demonstrated an ultracompact, ultrafast, nanoplasmonic based electron source that makes use of magnetostatic and physical filtering to generate attosecond keV electron packets. By varying the strength of the applied magnetic field or modification of the parameters of an aperture placed in front of the detector, both the kinetic energy of the electrons and the duration of the electron packet can be tailored. This compact attosecond keV electron source opens the pathway for time-resolved electron microscopy of physical, biological, and chemical processes with sub-nanometer and sub-femtosecond resolution such as real-time imaging of the formation and breaking of chemical bonds, imaging of protein folding, and imaging of intra-atomic processes. Further applications include ultrafast electron diffraction experiments, ultrafast electron crystallography experiments, and seeding of ultrafast nanoscale electron accelerators.

## Methods

### Three-dimensional electromagnetic simulation

The electromagnetic fields are calculated through the Yee-cell finite-difference-time-domain (FDTD) method^26^ utilizing realistic material parameters for the Ag film and silica conical lens. To reduce the computational requirements, the conical lens is simulated with a base diameter of 10 μm, which is larger than the laser spot size used. Symmetric boundary conditions are utilized in the *x* and *y* directions with Berenger PML boundary conditions^27^ in the *z* direction. The conical lens is placed 5μm away from the boundary in the *x,y* direction and 8μm away in the *z* direction. Furthermore, the source is placed 2.5μm back from the lens. The 50 nm Ag film is simulated with a random surface roughness of 2.8 nm rms (Ref. 25). The spatial electric field profile of the incident radially polarized laser beam is given by the following:


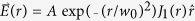
, where *A* is amplitude, *r* is the radial distance from the center of the beam, *w*_*0*_ is the beam waist, J_1_ is the first order Bessel function, and 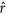 is a unit vector directed in the radial direction. For the linear polarization configuration, the spatial electric field profile is that of a standard Gaussian laser beam polarized in the x direction: 
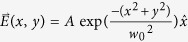
. For both configurations, the spatial electric field profiles are modulated in time by a 5 fs temporal Gaussian pulse having a central wavelength of 800 nm.

### Particle tracking simulation

To simulate the interaction of electrons with the electromagnetic fields of the conical surface plasmon (SP) wave, particle tracking code is implemented that tracks the particles through both space and time. Electrons are randomly placed (spatially and temporally) at the vacuum surface of the Ag film and are given a statistical weight that is proportional to the cube of the electric field intensity at their spatial and temporal generation point. Subsequently, the electrons are tracked through their interaction with the SP field via the nonrelativistic equation of motion 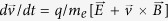, where 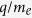
is the electron charge to mass ratio, 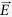 is the electric field, 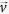 is the electron velocity, and 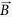 is the magnetic field. The electric and magnetic fields are taken from the FDTD calculations. By stepping in time through these fields, the electrons’ trajectories can be tracked in space and time. The static magnetic field utilized for filtering the electrons is introduced by appending a static magnetic field term to the equation of motion. To characterize the effect of the aperture on the electrons arriving at the detector, the aperture parameters *d, t,* and *l* are swept to select exemplary sets of parameters. The diameter and thickness of the aperture act to limit the acceptance angle of the aperture. From the parametric sweeps, representative electron packets are chosen based on their kinetic energies and temporal lengths. For all simulations, the detector is placed a fixed 50 μm away, in the + *y* direction (Fig. 1), from the tip of the conical lens. A total of 825,000 electrons are considered for each different magnetic field strength.

## Additional Information

**How to cite this article**: Greig, S. R. and Elezzabi, A. Y. Generation of attosecond electron packets via conical surface plasmon electron acceleration. *Sci. Rep.* **6**, 19056; doi: 10.1038/srep19056 (2016).

## Figures and Tables

**Table 1:**
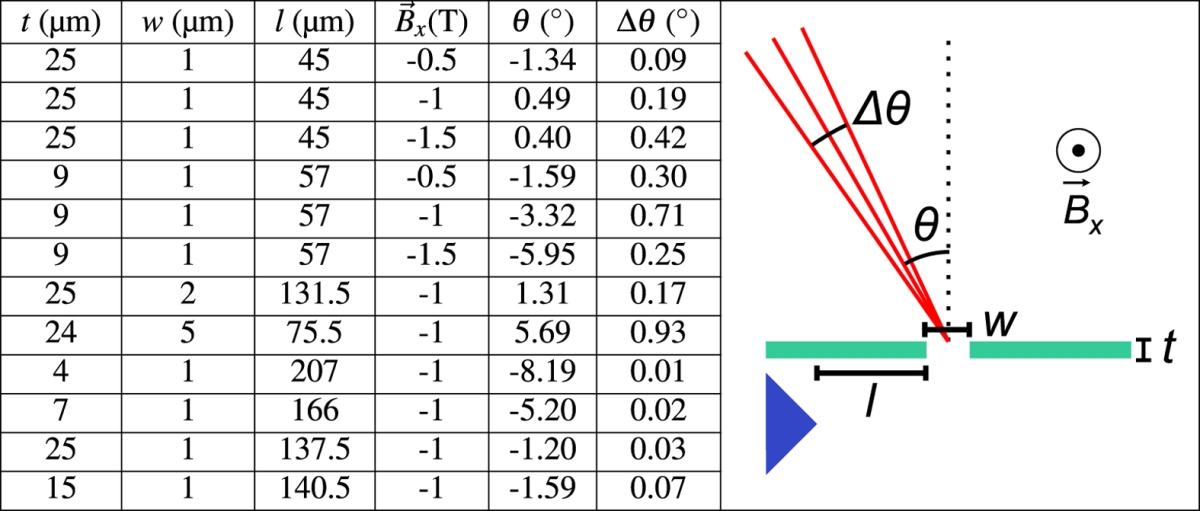
Electron packet angular spread after leaving the aperture.

*θ* is the central angle of the electrons and Δθ is the angular spread.
